# Electrical Bioimpedance Spectroscopy on Acute Unilateral Stroke Patients: Initial Observations regarding Differences between Sides

**DOI:** 10.1155/2015/613247

**Published:** 2015-10-18

**Authors:** Fernando Seoane, Seyed Reza Atefi, Jens Tomner, Konstantinos Kostulas, Kaj Lindecrantz

**Affiliations:** ^1^School of Technology and Health, KTH-Royal Institute of Technology, Alfred Nobels Allé 8, 14152 Huddinge, Sweden; ^2^School of Engineering, University of Borås, Allégatan 1, 50190 Borås, Sweden; ^3^Department of Neurology, R54, Karolinska University Hospital, Huddinge Unit, 141 86 Stockholm, Sweden; ^4^Department of Clinical Neuroscience, Neuro-Angiological Research Center, Karolinska Institute, Karolinska University Hospital, Huddinge, Sweden; ^5^Department of Clinical Science, Intervention and Technology, Karolinska Institute, 46 141 86 Stockholm, Sweden

## Abstract

*Purpose*. Electrical Bioimpedance Cerebral Monitoring is assessment in real time of health of brain tissue through study of passive dielectric properties of brain. During the last two decades theory and technology have been developed in parallel with animal experiments aiming to confirm feasibility of using bioimpedance-based technology for prompt detection of brain damage. Here, for the first time, we show that electrical bioimpedance measurements for left and right hemispheres are significantly different in acute cases of unilateral stroke within 24 hours from onset.* Methods*. Electrical BIS measurements have been taken in healthy volunteers and patients suffering from acute stroke within 24 hours of onset. BIS measurements have been obtained using SFB7 bioimpedance spectrometer manufactured by Impedimed ltd. and 4-electrode method. Measurement electrodes, current, and voltage have been placed according to 10–20 EEG system obtaining mutual BIS measurements from 4 different channels situated in pairs symmetrically from the midsagittal line. Obtained BIS data has been analyzed, assessing for symmetries and differences regarding healthy control data.* Results*. 7 out of 10 patients for Side-2-Side comparisons and 8 out 10 for central/lateral comparison presented values outside the range defined by healthy control group. When combined only 1 of 10 patients exhibited values within the healthy range.* Conclusions*. If these initial observations are confirmed with more patients, we can foresee emerging of noninvasive monitoring technology for brain damage with the potential to lead to paradigm shift in treatment of brain stroke and traumatic brain damage.

## 1. Introduction

Stroke is the third cause of death worldwide [1], killing more than five million people annually. However, the devastating consequences of stroke are not limited to these deaths. The consequences also include neurological dysfunction for another approximately five million people, thousands of millions of € in cost for emergency care, rehabilitation, and loss of productivity from those patients with permanent disabilities [2, 3].

There are US Food and Drug Administration (FDA) approved rescue therapies, such as recombinant tissue plasminogen activator rt-PA, for the 80–85% of the strokes that are ischemic, but the effectiveness of this treatment is significantly dependent on the time from onset to treatment [4].

Given the contraindication of rt-PA to patient with hemorrhagic stroke, 15–20% of the cases, proper identification of type of stroke is paramount prior to the initiation of therapy. Therefore successful treatment of stroke requires prior differentiation between ischemic and hemorrhagic damage. Unfortunately a correct differential diagnosis requires access to medical imaging modalities like CT and MRI involving processes that significantly increase the time door-to-needle, consequently delaying the initiation of treatment [5].

Electrical bioimpedance (EBI) technology has been used clinically and experimentally since the 1930s for assessment of several different physiological and pathophysiological mechanisms at cell, tissue, organ, and whole body level [6, 7]. Both applications of continuous single frequency measurements [8–21] and bioimpedance spectroscopy analysis (BIS) are well established in clinical practice [22–27] and their development for new application continues.

The use of EBI for the assessment of brain damage has been investigated from the 1950s [28] and during the last decade several authors have been proposing BIS as a potential useful noninvasive diagnostic tool for assessment of brain damage [29–36].

Recently Atefi et al. [37, 38] showed that it is possible to find differences in BIS measurements on patients having had a stroke. This paper presents clinical observations that suggest that stroke yields changes in cerebral BIS already in the early phases of stroke onset.

## 2. Methods

### 2.1. Bioimpedance Measurements

**Figure 1:**
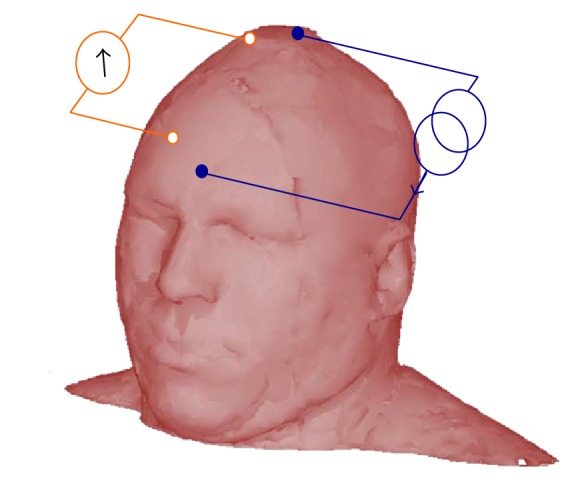
Tetrapolar method for noninvasive EBI measurements. One pair of electrodes is used to stimulate the head with the measurement current and another pair of electrodes senses the resulting voltage difference between the sensing electrodes.

Bioimpedance spectroscopy (BIS) measurements were collected using the four-electrode technique [39] where a pair of silver EEG electrodes is used to inject alternating electrical current into the head and a separate pair of electrodes is used to measure the corresponding voltage difference on the surface of the head; see Figure 1.

**Figure 2:**
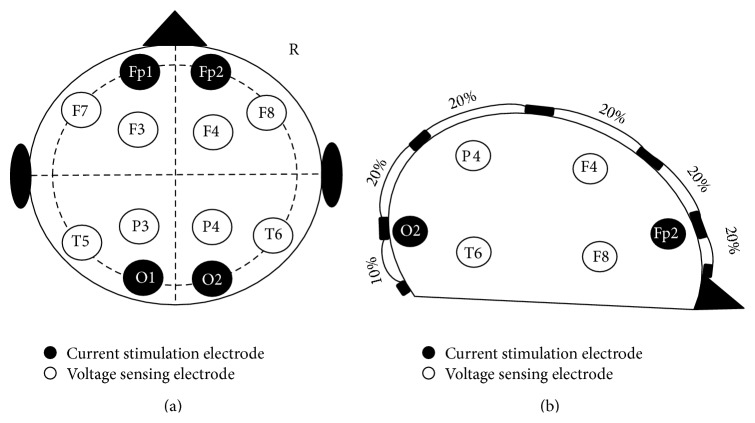
10–20 international EEG landmarks. Indicates where the stimulating electrodes, filled circles, and the sensing electrodes, hollow circles, were placed for performing the BIS measurement for each of the four measurement channels lateral and central for right and left side.

The transfer function between the current injecting pair and voltage sensing pair will provide the transfer impedance [40]. In this study current injecting and voltage sensing positions are labeled according to the international 10–20-electrode placement system. BIS from the left hemisphere had current injecting pair placed on frontal and occipital lobes of the left hemisphere (Fp1-O1) forcing the current to propagate into the left hemisphere and the voltage difference was measured in lateral and central areas. Lateral voltage difference was measured between the left frontal and temporal lobes (T6-F8) and the central voltage difference was recorded between left parietal and frontal lobes (P4-F4). Identical measurements on the right hemisphere had Fp2-O2 for current pair, T5-F7 for lateral voltage difference, and P3-F3 for central voltage difference; see Figure 2.

For each patient, the skin on the electrode positions was prepared scrubbing with abrasive paste (EleFix) and then the silver EEG electrodes dipped in electroconductive paste (Elefix) were placed by an EEG expert to ensure accurate electrodes positioning for each subject.

### 2.2. BIS Instrument and Transfer Impedance Estimation

BIS was recorded by a commercial bioimpedance spectrometer SFB7 manufactured by Impedimed Ltd. The SFB7 records BIS at 256 logarithmically spaced frequency points in the range 3.096–1000 kHz by applying a sinusoidal current with constant RMS amplitude of 200 *μ*A at each frequency point and calculating the transfer impedance at that frequency as the ratio between the current pair and voltage sensing pair. This way SFB7 calculates 256 complex impedance values known as impedance spectra. Impedance spectra from central left volume are labeled as *Z*_CL_(*ω*) and the one from right volumes is labeled as *Z*_CR_(*ω*). Similarly *Z*_LL_(*ω*) and *Z*_LR_(*ω*) indicate lateral left and lateral right impedance spectra. This way the resistance spectrum, the* real part of the impedance, will be *denoted as *R*_CL_(*ω*), *R*_CR_(*ω*) for left and right central measurements, respectively, and *R*_LL_(*ω*) and *R*_LR_(*ω*) for left and right lateral measurements.

### 2.3. BIS Measurements on Controls and Patients

*Healthy Population.* Three male subjects between 29 and 59 years of age and without any previous neurological complication were enrolled to Salhgrenska University Hospital. The Ethics Regional Committee of Gothenburg approved clinical protocols for these measurements. 

*Stroke Population.* Ten patients, five female and five male (mean age 73 ± SD 14), were enrolled from the Stroke Ward at Karolinska University Hospital in Huddinge. The measurements were obtained within the first approximated 24 hours from onset. The precise onset time was not known in several patients, due to onset during sleep. The NIH score was 1–19 (mean 5,9 ± SD 5,5). Four patients had hemorrhagic stroke, one of which bled after administration of rt-PA. The bleedings were located centrally in two patients (one in the right putamen and one in the right medial occipital lobe) and laterally in the other two (one in the left operculum and one in the lateral part of the right occipital lobe). The six ischemic strokes were of various sizes and location. Three were large lesions in the territory supplied by the left posterior artery. Two were infarctions in the region supplied by the right media artery. One patient (patient I) had small infarctions in the left nuclei caudati and putamen not visible on the initial CT scan. Patient details are summarized in Table 1. The Ethics Regional Committee of Stockholm approved all of the clinical protocols. 

*BIS Measurements*. Twenty (20) consecutive *Z*_LL_, *Z*_LR_, *Z*_CL_, and *Z*_CR_ spectroscopy measurements were recorded from each healthy/patient and the mean resistance of each set was calculated at each measured frequency, for example,* mean of 20 resistance spectra for each set* was calculated and labeled as *R*_LL_(*ω*), *R*_LR_(*ω*), *R*_CL_(*ω*), and *R*_CR_(*ω*), respectively.

### 2.4. Symmetry Analysis and Visualization of BIS Data

**Figure 3:**
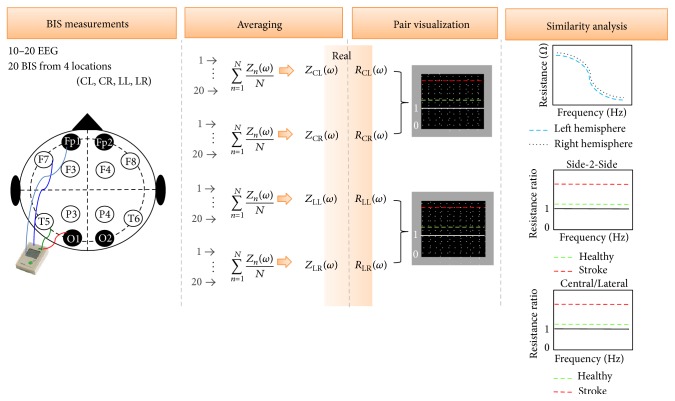
BIS recording analysis diagram. 20 consecutive complex BIS measurements are recorded from 4 different volumes of each healthy/stroke (*Z*_CR/ZCL_(*ω*) and *Z*_LR/LL_(*ω*)), and the real parts (*R*_CR/CL_(*ω*) and *R*_LR/LL_(*ω*)) are analyzed by pair evaluating symmetry and similarities.

Patients admitted with acute stroke, that is,* less than 24 hours*, underwent either CT or MR scans as part of routine clinical procedures. Following the scan, after obtaining the written consent, BIS recordings were collected from each patient and transferred to MATLAB for data analysis; see Figure 3 for a descriptive diagram.

The core hypothesis for comparison of healthy and damaged brain is that identical volumes from the two hemispheres of healthy subjects should have minimal differences in the impedance spectra between hemispheres, while in cases with unilateral brain injury this should not be the case as the electrical properties of one hemisphere, that is,* in the damaged area* is changed compared to its undamaged counterpart.

In order to observe any differences between healthy and damaged brains, two straightforward approaches have been used:

 1st is Side-2-Side ratio: the ratio between symmetrically located BIS measurements has been calculated for the resistance spectrum, that is,* one ratio has been obtained for the left and right lateral measurements and one for the left and right central measurements*.
 2nd is central/lateral ratio: the ratio between central and lateral BIS measurements has been obtained for the resistance spectrum; therefore again 2 ratios, one for left and one for right, are obtained from each subject.

In both cases, the ratio function obtained from the stroke patient measurements has been plotted together with the ratio obtained from the healthy control measurements.

## 3. Results

All the BIS measurements obtained for the healthy control and the stroke patient group are plotted in the Appendix. In this section for improving the readability, only results obtained from the specific comparison required to support the symmetric analysis are presented (Table 2 shows overall comparison for all patients and ratios).

### 3.1. Healthy Control Cases

**Figure 4:**
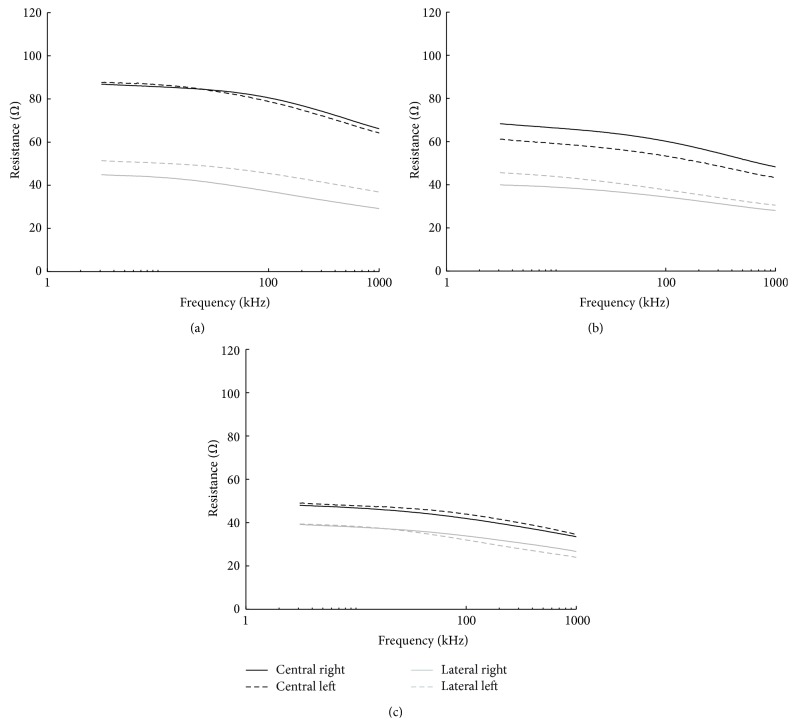
Healthy EBI Spectra. Lateral and central bioimpedance spectrum obtained for each of the 3 healthy volunteers in plots (a), (b), and (c), respectively. Central and lateral bioimpedance spectra are plotted in pairs.

The spectral plots included in Figure 4 reproduce the BIS measurements obtained for the 3 control healthy subjects. Observe that the spectra for right with continuous trace (−) and left with dashed trace (- -) are basically paired according to location. In the same plots it is possible observe that the central measurements, with dark trace, present a larger impedance magnitude than the measurements obtained from the lateral locations with light trace.

**Figure 5:**
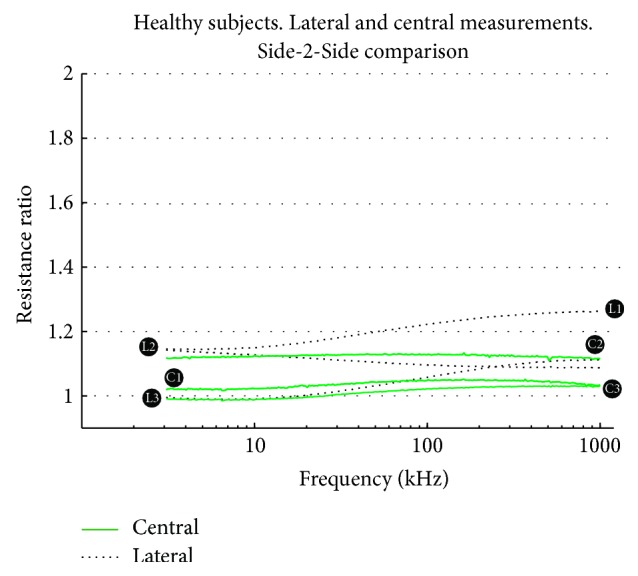
Side-2-Side ratio comparison in healthy EBI spectra. Resistance ratio obtained for lateral and central BIS measurements for the control cases.

In Figure 5 it is possible to observe that the Side-2-Side ratio obtained from the symmetrically located BIS measurements on healthy measurements is close to 1. The ratio obtained for central measurements, continuous trace (−), exhibit a smaller dispersion than the ratio exhibit by the lateral measurements with dotted trace (•). Observe that while the deviation from the unity at low frequency is very small, it increases with frequency in some cases.

### 3.2. Side-2-Side Symmetrical Comparison

**Figure 6:**
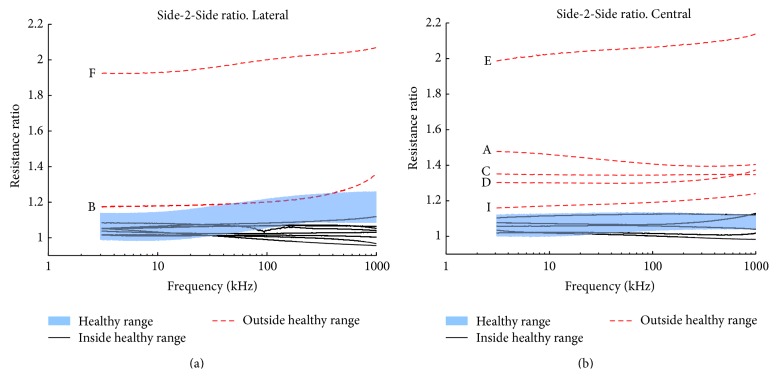
Side-2-Side ratio resistance comparison. Resistance ratio obtained for lateral BIS measurements in (a) and central in (b). The range observed in the control cases for the Side-to-Side ratio is denoted by the shadowed region near the value 1. Notice that the central ratio from patients A, C, D, E, and I in (b) and the lateral ratios from B and F in (a) are outside the healthy range.

In Figure 6(a), when comparing the Side-to-Side ratio obtained for the central BIS measurements performed on all the patients, it is shown that the ratio in at least 5 of the patients, dashed thick trace (- -), exhibits a ratio noticeable larger than the unity and the ratio values for the control measurements denoted by the shadowed area. There are another 5 patients reporting ratio values, continuous thin dark trace (−), within the range exhibited by the control healthy group.

When looking at the Side-to-Side comparison for the lateral measurements, the plot in Figure 6(b) shows that only in 2 cases, dashed trace (- -), the ratio values are outside the shadowed area. For the other cases, plotted with continuous thin dark trace (−), the ratio values fall inside the limits exhibited by the control group, blue shadowed area.

### 3.3. Central over Lateral Comparison

**Figure 7:**
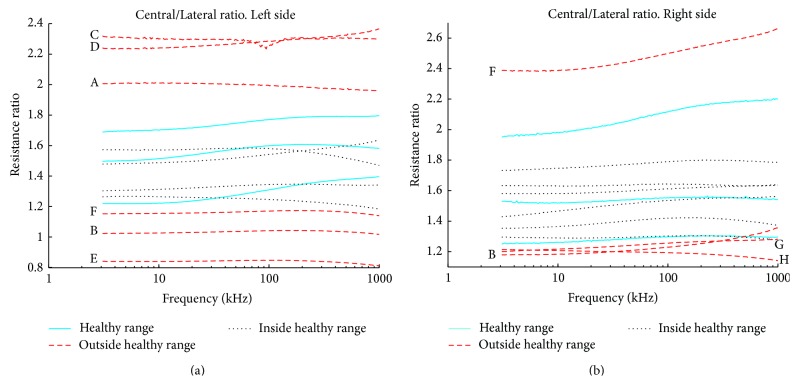
Central over lateral ratio comparison. Resistance ratios obtained from central divided lateral BIS measurements for left in (a) and right in (b) are shown. The range observed in the control cases for the Side-to-Side ratio is denoted by the shadowed region. Notice that the resistance ratio from patients A to F in (a) and B, F, G, and H, in (b) are outside the healthy range.

In Figure 7 the spectral plots representing the ratio are obtained from comparing central and lateral measurements in each subject. The ratio plots obtained for the left side, Figure 7(a), indicate that the ratio values obtained from healthy measurements, range between 1.2 and 1.8 approximately. In the plot it is possible to see that the ratio values from 6 patients (A–F), dashed thick trace (- -), produce ratio values clearly below or above the control range. Patients (G–J) present ratio values within the healthy range, continuous thin dark trace (−). For the right side, in Figure 7(b), we can observe that there are 4 patients (B, F, G, and H) with ratio values, dashed trace (- -), above or below the values 1.25–2.20 defining the healthy range.

## 4. Discussion

### 4.1. Side-2-Side Symmetries

The main working hypothesis is that ideally, identical symmetric cerebral hemispheres will exhibit similar impedance spectra, given that the measurements are taken with electrode arrangements using anatomical landmarks from the midsagittal plane.

In reality the cerebral hemispheres are not identical, and the brain is not located within the cranium in perfect symmetry with reference to the 10–20 landmarks. Moreover, it is likely that the electrophysiology technician has certain variability in the placement of electrodes. Such source of error will likely influence in such kind of comparison but, in any case, despite the potential sources of error that might influence the different spectral BIS measurements, it is possible to see that the measurements on healthy volunteers are remarkably similar when paired by symmetry with respect to the midsagittal line as shown in Figures 4 and 5.

The obtained results show that the majority of the patients, 7 out of 10, exhibit a ratio larger than the ratios obtained from the healthy.

In general it is reasonably expected that a lesion in any of the cerebral hemispheres should contribute significantly to modify the current density distribution across the brain and produce noticeably different BIS measurements between channels that would otherwise produce similar, quasi-identical, BIS measurements. A potential exception would be centrally located lesions that could potentially preserve the symmetry, but in this study all lesions were laterally located.

### 4.2. Other Expected Relationships between BIS Measurements

Another observation that can be done from the BIS measurements performed on healthy volunteers plotted in Figure 4 is that the magnitude of the resistance spectra from the central measurements is larger than lateral measurements. It is naturally expected with the measurement setup use in the study that in healthy brain the BIS measurements recorded from the central locations should be larger than the measurements obtained from the laterally located channels, but in addition it is also naturally expected that the central to lateral relations should have a given maximum value as well, delimiting a healthy range.

When comparing the BIS measurements regarding the central and lateral channels included in the Appendix and the ratios plotted in Figure 7, it is possible to see that in 8 out of 10 patients the values of the obtained ratios are below or above the values obtained for the corresponding healthy control cases.

### 4.3. Summary of Observations

When analyzing the results from all the comparisons, it is possible to see that up to 9 patients present at least one or more resistance ratios different from those calculated from the healthy control. The majority of the patients, 6 out 10, exhibit 2 or more ratios out from the healthy range. Taking a closer look at those other 4 patients, G–J, we realized that in each of them the location of the brain lesion was at the same level or below the third ventricle or the putamen. Lesions at such depth might require electrodes to be located in specific positions to force the current measurement to flow through deeper structures.

### 4.4. Injury Depth, Electrode Location, and Impedance Sensitivity

In a BIS measurement, the measurement current is distributed through the volume according to the conductivity of the constituting tissue but it will also depend on the location of the different tissue structures in relation to the position of electrodes. With the position of both voltage and sensing electrode used in this study, it is known that the current density and the lead fields through deeper tissue structures are significantly smaller [41] and therefore it will have less contribution to the total measured impedance. Therefore the altered conductivity of the brain injury might not need to produce a noticeable difference in the BIS measurement.

### 4.5. Relation to Related Work

The value of these initial observations is that they show for the first time that stroke, already in its early phases, modifies the electrical properties of the brain and differences can be observed through electrical bioimpedance spectroscopy measurements of the head in acute unilateral stroke patients. This way validates the theories and hypothesis presented in [30, 42–45] and complementing simulation results reported recently in [46]. Where in an analogous manner of comparing hemispheric left and right sides over using conductivity images obtained with Electrical Impedance Tomography at a single frequency for each hemisphere should not present any differences in healthy subjects but in patients with unilateral stroke the conductivity images showed observable differences, according to the simulations.

### 4.6. Brain Damage Identification and Potential Usage of EBCM

The ultimate goal of any novel diagnostic support system for early detection of stroke would be not only to detect the damage but also to differentiate between ischemic and hemorrhagic damage. From that perspective, the preliminary analysis performed in this study indicates that no differentiation between stroke patients with ischemia or hemorrhage could be done on the basis of the recorded BIS measurements. Given the limited number of subjects and considering the many differences among the stroke lesions regarding location and type, clear distinctive differences in the BIS data to allow any kind of type differentiation were not expected in this first attempt of studying BIS data obtained from stroke acute patients.

Differentiation of type of stroke damage based on BIS technology, if possible, would provide bioimpedance with a paramount importance in the stroke triage protocol, that is, not the only way that BIS could be useful when caring for stroke patients. A diagnostic tool available to the Emergency Medicine Services personal onboard of the ambulance that could perform quick and direct assessments about the existence of brain damage would provide significant support to paramedics when deciding to prioritize primary stroke centers against closer nonprimary stroke centers like in stroke triage protocols like in [47].

## 5. Conclusion and Future

It was known that patients with chronic stroke lesions exhibited differences in BIS measurements [37, 38]; now these initial observations confirm that changes can be noticed from the initial phases, making bioimpedance spectroscopy a potential candidate to base the development of a novel diagnosis support tool for prompt detection of stroke attack. To remove the word potential requires an additional validation that measurements of bioimpedance can indeed detect brain damage within the first hour from onset. In this regard, efforts led by the Royal Institute of Technology, a project proposal has been submitted to the EU H2020 funding program, in which, among other things, the detection performance with time will be evaluated.

As it is known in stroke care* Time is Brain* because early treatment produces better outcome [48]. Therefore to reduce the door-to-needle time when caring for brain damage patient is very important and for that detection of ongoing brain damage is a significant feature but, to eliminate the door-to-needle time when treating acute stroke, it is necessary not only to detect the on-going stroke but also to differentiate the type in the very first stages of the care process, for example,* in the ambulance during acute transport*.

While the replacement of CT technology by BIS technology in the stroke triage protocol seems far away in time and slightly unrealistic at this point, to envision a tool for assessment of brain damage for supporting first responders at the earliest stage of a stroke triage protocol should guide any development in the field of Electrical Bioimpedance Cerebral Monitoring in the near future.

A priori EBI technology fulfills all the requirements to allow the development of such BIS-based tool that is,* portable, noninvasive, affordable, and compact in size*. Although these initial observations on clinical data are very encouraging and validate years of theoretical, simulation, and animal studies yet extensive clinical, biomedical engineering and more theoretical research must be executed towards the development of such diagnostic tool for prompt detection of brain damage. Among them, we can identify targeting the acquisition of reference values for controls and BIS characterization of healthy brain, the study of the several factors influencing the bioimpedance of different types of lesions specifically including size and location of the lesion. Moreover future investigations should include the use of different arrangements of electrodes, to target deeper structures; for such purpose 3D-simulations with realistic anatomic models would be especially helpful.

## Figures and Tables

**Figure 8:**
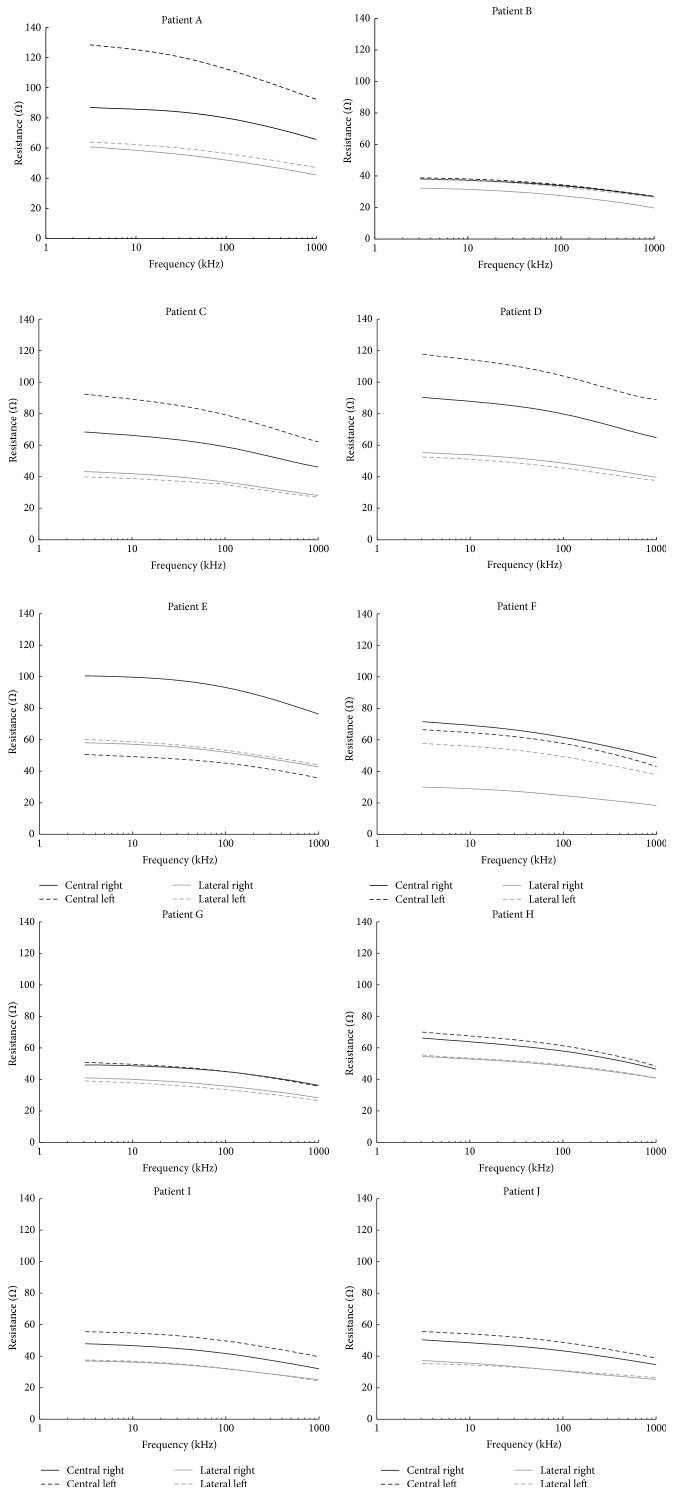
Patient group.

**Table 1:** Patient details.

Patient	Gender	Age	Lesion	Location	NIH Day 1
A	M	82	Ischemic stroke	Right MCA	19
B	W	44	Ischemic stroke	Left PCA	1
C	W	70	Ischemic stroke	Left PCA	6
D	W	81	Ischemic stroke^*∗*^	Right PCA	4
E	W	93	Ischemic stroke	Right MCA	8
F	M	82	Hemorrhagic stroke	Lobar Left	1
G	M	66	Hemorrhagic stroke	Deep Right	12
H	W	84	Ischemic stroke	Left PCA	5
I	M	56	Ischemic stroke	Deep Left	1
J	M	71	Hemorrhagic stroke	Lobar Right	2

^*∗*^See Appendix.

**Table 2:** Overall comparison for all patients and ratios.

	Patient									
	A	B	C	D	E	F	G	H	I	J
Ratio comparison										
Side-2-Side central	*✗*		*✗*	*✗*	*✗*				*✗*	
Side-2-Side lateral		*✗*				*✗*				
Central/Lateral left	*✗*	*✗*	*✗*	*✗*	*✗*	*✗*				
Central/Lateral right		*✗*				*✗*	*✗*	*✗*		

## Appendix

### BIS Measurement Plots for Stroke Patients

See Figure 8.
